# The Draft Report by the Institute for Quality and Efficiency in Healthcare Does Not Provide Any Evidence That Graded Exercise Therapy and Cognitive Behavioral Therapy Are Safe and Effective Treatments for Myalgic Encephalomyelitis/Chronic Fatigue Syndrome

**DOI:** 10.3390/diseases11010011

**Published:** 2023-01-16

**Authors:** Mark Vink, Alexandra Vink-Niese

**Affiliations:** 1 Independent Researcher, 1096 HZ Amsterdam, The Netherlands; 2 Independent Researcher, 30159 Hannover, Germany

**Keywords:** chronic fatigue syndrome, cognitive behavioral therapy, exercise, long COVID, myalgic encephalomyelitis, post-acute COVID-19 syndrome, post-infectious disorders

## Abstract

The German Institute for Quality and Efficiency in Healthcare (IQWiG) recently published its draft report to the government about myalgic encephalomyelitis/chronic fatigue syndrome (ME/CFS). The IQWiG concluded that graded exercise therapy (GET) and cognitive behavioral therapy (CBT) should be recommended in the treatment for mild and moderate ME/CFS based on two CBT and two GET studies. In this article, we reviewed the evidence used by IQWiG to support their claims, because their conclusion is diametrically opposed to the conclusion by the British National Institute for Health and Care Excellence (NICE) in its recently updated ME/CFS guidelines. Our analysis shows that the trials IQWiG used in support suffered from serious flaws, which included badly designed control groups; relying on subjective primary outcomes in non-blinded studies; alliance and response shift bias, including patients in their trials who did not have the disease under investigation, selective reporting, making extensive endpoint changes and low to very low adherence of treatments. Our analysis also shows that the report itself used one CBT and one GET study that both examined a different treatment. The report also used a definition of CBT that does not reflect the way it is being used in ME/CFS or was tested in the studies. The report noted that one study used a wrong definition of post-exertional malaise (PEM), the main characteristic of the disease, according to the report. Yet, it ignored the consequence of this, that less than the required minimum percentage of patients had the disease under investigation in that study. It also ignored the absence of improvement on most of the subjective outcomes, as well as the fact that the IQWiG methods handbook states that one should use objective outcomes and not rely on subjective outcomes in non-blinded studies. The report concluded that both treatments did not lead to objective improvement in the six-minute walk test but then ignored that. The report did not analyze the other objective outcomes of the studies (step test and occupational and benefits status), which showed a null effect. Finally, the report states that the studies do not report on safety yet assumes that the treatments are safe based on a tendency towards small subjective improvements in fatigue and physical functioning, even though the adherence to the treatments was (very) low and the studies included many patients who did not have the disease under investigation and, consequently, did not suffer from exertion intolerance contrary to ME/CFS patients. At the same time, it ignored and downplayed all the evidence that both treatments are not safe, even when the evidence was produced by a British university. In conclusion, the studies used by the report do not provide any evidence that CBT and GET are safe and effective. Consequently, the report and the studies do not provide any support for the recommendation to use CBT and GET for ME/CFS or long COVID, which, in many cases, is the same or resembles ME/CFS, after an infection with SARS-CoV-2.

## 1. Introduction

The German Institute for Quality and Efficiency in Healthcare (IQWiG) recently published its draft report to the government about myalgic encephalomyelitis/chronic fatigue syndrome (ME/CFS) [1]. IQWiG noted that ME/CFS has been classified as a neurological disease in the international classification of diseases (ICD) since 1969 (in German: “ME/CFS wurde schon 1969 in der ICD als neurologische Krankheit aufgenommen” p. 28) and concluded that ME/CFS is characterized by post-exertional malaise (PEM), as well as various other symptoms such as severe and persistent fatigue, pain, sleep disturbances and cognitive disorders. It defined PEM as the worsening of symptoms after only slight physical or mental activities that can last for days or weeks. They also noted that it seems plausible to assume that there may be an increase in ME/CFS prevalence because of the severe acute respiratory syndrome coronavirus 2 (SARS-CoV-2) that caused the coronavirus disease 2019 (COVID-19) epidemic, since, according to them, some post-COVID patients meet the ME/CFS diagnostic criteria.

It recommended graded exercise therapy (GET) and cognitive behavioral therapy (CBT) for patients with mild-to-moderate ME/CFS, because their benefit assessments based on two CBT and two GET studies found a hint of an indication (weakest reliability) for short and medium-term benefits of both CBT and GET in comparison to standard medical care (SMC). This benefit was not found at a longer follow-up.

Their conclusion, however, is diametrically opposed to the conclusion by, for example, the prestigious American Institute of Medicine (IOM, now the National Academy of Medicine) in 2015 [2], the Dutch Health Council in 2018 [3] and the British National Institute for Health and Care Excellence (NICE) in 2021 [4] that there is no effective treatment for ME/CFS. It is also diametrically opposed to several reviews [5,6,7,8], which concluded that recommendations for CBT and GET for ME/CFS are based on flawed and biased evidence produced by studies that are of poor methodological quality and ignore the absence of objective improvement in their own studies.

In this article, we will review the IQWiG report and analyze the three trials they used as evidence regarding the safety and efficacy of CBT and GET to see if there is any merit in it or if IQWiG should have come to a different conclusion. This is especially important, because, currently, millions of patients are falling ill with post-COVID syndrome after an initial, often mild, infection with SARS-CoV-2. Post-COVID syndrome is often referred to as long COVID, and in many cases, this is the same or resembles ME/CFS [9]. A study by Davis et al. found that 89% of long COVID patients suffered from PEM, 88% from cognitive problems and many patients also suffered from other ME/CFS symptoms [10].

## 2. Requirements for Evidence from Studies for ME/CFS to Be Scientifically Valid

According to, for example, the BRANDO project (*Bias in Randomised and Observational studies*) [11], which, amongst others, included Stanford Professor Ioannidis, it is important that “as far as possible, clinical and policy decisions should not be based on trials in which blinding is not feasible and outcome measures are subjectively assessed”, because the lack of blinding is “associated with an average 13% exaggeration of intervention effects.” “Therefore, trials in which blinding is not feasible should focus as far as possible on objectively measured outcomes” (p. 23, 45). Consequently, CBT and GET studies, which are non-blinded by definition, should use objective primary outcomes alone or in combination with subjective ones to avoid the erroneous interference of efficacy in its absence. 

On top of that, there is a low correlation between objective and subjective activity measurements [12] not only in chronically ill but also in healthy people [13]. CBT researchers from the Netherlands, which included Professor Bleijenberg, who is one of the researchers of one of the studies [14] used by the report [1], concluded “that one has to be very careful with using self-report questionnaires as measures for actual activity level: [as] none of the self-report questionnaires had strong correlations with the Actometer” (p. 670 [14]). Additionally, the reanalysis of the amended Cochrane exercise review [15] found that objective outcomes from three CBT and GET trials for ME/CFS confirmed the unreliability of the subjective outcomes in non-blinded studies, as shown by the following examples:

- In Jason et al., 2007 [16], there was a substantial difference in the subjective physical functioning scores at baseline between the exercise and control groups, yet, objectively, there was not (six-minute walk test or 6MWT);
- In Moss-Morris et al., 2005 [17], after GET, physical functioning subjectively improved by 30%, yet, objectively, deteriorated by 15% (CPET);
- In the PACE trial, the released individual participant data showed that 20% of participants whose physical functioning improved subjectively had deteriorated objectively (6MWT) [18,19,20].

The IQWiG methods guidance handbook [21] states the following about this problem. The value of patient-reported outcomes (PROs) in non-blinded studies is limited because of the subjective nature of them. (In German, “Da Angaben zu PROs aufgrund ihrer Natur subjektiv sind, sind offene, d. h. nicht verblindete Studien in diesem Bereich nur von eingeschränkter Validität” p. 61). The same handbook also states that non-blinded studies should, as far as possible, rely on objective endpoints, because subjective ones can be influenced by the person collecting them. (In German, “Falls eine verblindete Zielgrößenerhebung nicht möglich ist, sollte ein möglichst objektiver Endpunkt gewählt werden, der in seiner Ausprägung und in der Stringenz der Erfassung so wenig wie möglich durch diejenige Person, die den Endpunkt (unverblindet) erhebt, beeinflusst werden kann” p. 171).

A key principle of a randomized controlled trial (RCT) to ensure a fair comparison is that groups should be similar with respect to all factors that might affect the outcome, besides the intervention, including the number of treatment sessions, to ensure a fair comparison [22] but, also, to make sure that an RCT is ‘internally valid’, which refers to the extent that the outcome for a trial can be attributed to the experimental treatment and not to any alternative explanation, such as the natural course of the target problem [23]. Yet, in a waitlist control group, patients do not get any treatment from (a doctor from) that study. In a standard medical care (SMC) control group, the number of treatment sessions is usually a lot less and a session is usually a lot shorter than in the treatment group. Consequently, a waitlist control group and a SMC control group do not adequately correct for the placebo effect, regression to the mean and other forms of biases and confounding factors. In short, control conditions may create threats to the internal validity by influencing the outcome expectancies (p. 276 [23]).

The IQWiG methods guidance handbook [21] states the following about this problem. A randomized controlled trial must be designed in such a way that a difference between the intervention and control groups can only be traced back to a single influencing variable—the tested intervention. (In German, “Grundlage einer Nutzenbewertung ist der Nachweis von Kausalität. Unverzichtbare Bedingung für den Nachweis von Kausalität ist ein vergleichendes Experiment, das so angelegt sein muss, dass ein Unterschied zwischen Interventionsgruppen—ein Effekt—nur auf eine einzige Einflussgröße—die geprüfte Intervention—zurückgeführt werden kann. Dieses Ziel macht für klinische Studien erhebliche Anstrengungen nötig, weil es zahlreiche unerwünschte Einflüsse gibt, die einen Effekt vortäuschen oder auch verbergen können (Verzerrung)” p. 10). 

Additionally, participants expect to get some form of treatment in return for taking part in a trial. Yet, in a no treatment, SMC or waiting list control group (WLC), they must attend several assessments without any direct benefit for themselves. These patients will be disappointed that they have been denied treatment benefits they anticipated from participation in a study. Assignment to those sort of control groups may strengthen participants’ beliefs that they will not improve, thereby reducing the chance of spontaneous improvement. On top of that, participants randomized to those sorts of control conditions may improve less than would be expected compared to participants not enrolled in a trial. Yet, researchers often assume that with no-treatment control designs, the absence of treatment equates with the absence of an effect. This assumption may threaten the internal validity, as participants randomized to WLC conditions may improve less than would be expected compared to participants not enrolled in a trial. Consequently, subjective baseline–follow-up differences cannot be assumed to be the natural history of what would have occurred in the absence of patients enrolling in the study. Therefore, using waitlist, SMC or no-treatment control conditions can lead to the overestimation of the effectiveness of a treatment [23]. Moreover, Janse et al. [24], who used a waiting list control group, noted that “the use of a waiting-list control [group] does not control for non-specific therapy factors and limits the external validity” (p. 116). 

A Cochrane review of non-pharmacological interventions for functional syndromes, including CFS (14 of the 21 RCTs used CBT) [25], and a recent meta-analysis of 33 RCTs of mindfulness-based interventions [26], both not only concluded that most benefits disappear when an active control group is used instead of a waitlist or no-treatment control group but, also, that there is evidence as well that the remaining effects are inflated by bias and multiple methodological concerns, including high drop-out rates and selective biases in sampling. In conclusion, neither a waitlist control group nor a SMC control group are properly controlled control groups. 

On top of that, a primary outcome should be clinically meaningful, relevant to the patient, clearly defined and measurable [22]. A systematic review by Haywood et al. [27] of patient-reported outcome measures (PROMs) in ME/CFS found poor quality in the reviewed PROMs, which included the often-used Chalder Fatigue Scale, with clear discrepancies between what is measured in research and which outcomes are relevant to patients. They also concluded that “the poor quality of reviewed PROMs combined with the failure to measure genuinely important patient outcomes suggests that high quality and relevant information about treatment effect is lacking” (p. 48). A systematic review by Whiting et al. [28] concluded in 2001 that subjective outcomes in ME/CFS are unreliable, because a “person may feel better able to cope with daily activities because they have reduced their expectations of what they should achieve, rather than because they have made any recovery because of the intervention. A more objective measure of the effect of any intervention would be whether participants have increased their working hours, returned to work or school, or [objectively] increased their physical activities” (p. 1366). 

Finally, according to the IQWiG report, the presence of PEM is a central diagnostic criterion [1]. Consequently, only participants who suffer from it should be included in studies. Alternatively, studies should present their results for participants with and without PEM separately. Studies that do not do this should be excluded, because including patients who do not have the disease under investigation might lead to “the erroneous interferences of improvement” in its absence (p. 363 [29]).

## 3. Analysis of the Evidence Relied upon by the IQWiG Report

The IQWiG report used the updated NICE guidelines from 2021 as its basis and identified a total of 85 randomized controlled trials (RCTs) of non-drug and drug-based therapies, yet noted that, in 77 of these studies, diagnostic criteria were used that did not require PEM, nor was the percentage of participants in those studies who suffered from PEM reported. The IQWiG concluded therefore that it is unknown to what extent patients with ME/CFS actually took part in these studies. The remaining eight studies investigated CBT, GET, pacing, the Lightning Process, vitamin D, valganciclovir and rituximab, which, in most studies, were compared to standard care. According to the report, there were only significant effects in favor of the interventions, CBT and GET, respectively, compared to standard care in two studies each. The report carried out separate benefit assessments for these two interventions, whereby it analyzed Janse et al. from 2018 [24] and the PACE trial by White et al. from 2011 [20] to assess the efficacy of CBT and the PACE trial [20] and the GETSET trial by Clark et al. from 2017 [30] to assess the efficacy of GET. 

### 3.1. Wrong Definition of CBT and Study Included That Did Not Examine Efficacy of CBT

The IQWiG report defined CBT in the following manner [1]. Cognitive behavioral therapy (CBT) describes a psychotherapy procedure that is based on the findings of empirical psychology—in particular, learning and social psychology. CBT assumes that disorders can be related to negative, unrealistic and distorted thought and behavior patterns. Accordingly, the aim of CBT is to change the dysfunctional cognitions, because then, the experiences, feelings and behaviors of a person change. For this purpose, CBT integrates various therapy methods, such as education about the symptoms and causes of the disorder, promotion of social skills, concrete behavioral exercises (e.g., development of positive activities and reduction of avoidance behavior) and cognitive restructuring. (In German: “CBT (kognitive Verhaltenstherapie). Die kognitive Verhaltenstherapie (CBT; cognitive behavioral therapy) beschreibt ein Psychotherapieverfahren, welches auf Erkenntnissen der empirischen Psychologie, im Speziellen der Lern- und Sozialpsychologie, beruht. CBT geht davon aus, dass Störungen mit negativen, realitätsfremden und verzerrten Denk- und Verhaltensmustern zusammenhängen können. Ziel einer CBT ist entsprechend eine Veränderung der dysfunktionalen Kognitionen, in deren Folge das Erleben, das Fühlen und das Verhalten einer Person sich verändern. Zu diesem Zweck integriert die CBT diverse Therapiemethoden, wie bspw. Aufklärung über die Symptome und Ursachen der Störung, Förderung der sozialen Kompetenzen, konkrete Verhaltensübungen (bspw. Aufbau positiver Aktivitäten und Reduktion Vermeidungsverhalten) und kognitive Umstrukturierung” p. 37). Yet, CBT for ME/CFS also contains an element of GET whereby participants need to “gradually increase the amount of activity you do each day” (p. 45), according to, for example, the CBT manual for participants from the PACE trial [31]. However, when patients do that, it triggers PEM, which might lead to relapses. It also means that the two studies that were used by the IQWiG report to recommend CBT examined a different form of CBT. Moreover, the study by Janse et al. from 2018 [24] did not examine the efficacy of face-to-face CBT. Instead, it tested the efficacy of two forms of internet-based CBT (“we tested the efficacy of iCBT with… guidance in a protocol-driven feedback condition. We also tested the efficacy of iCBT in which guidance was only given when patients asked for it, i.e., a feedback-on-demand condition” p. 112). Consequently, this study should have been excluded from the IQWiG analysis. 

### 3.2. Study Included That Did Not Examine Efficacy of GET

The IQWiG report used two studies to determine the efficacy of GET. However, it is unclear why the IQWiG report used the GETSET trial as evidence for the efficacy of GET, because the study itself states the following in their therapist’s manual about the treatment (Guided graded Exercise Self-hElp or GES) tested in their trial [32]. “The principles of the treatment [GES] are based on graded exercise therapy (GET), but *it is not GET* [italic by us] because GET is a therapy delivered by a physiotherapist face-to-face in a clinic setting” (p. 7). Consequently, this study should have been excluded from the IQWiG analysis. 

### 3.3. Bias

All three trials used by the IQWiG report [1] relied on subjective primary outcomes, even though they are non-blinded by definition. The unreliability of subjective outcomes in non-blinded trials was discussed earlier. Additionally, as noted earlier, “trials in which blinding is not feasible should focus as far as possible on objectively measured outcomes” (p. 23, 45). All three studies used by the IQWiG failed to do this. The IQWiG report acknowledges this problem when it states that “Blinding”/“lack of blinding” is always an aspect in the institute’s benefit assessments when assessing the risk of bias. In this report, for example, the PACE study was attested to have a high risk of bias. In the cross-endpoint overall consideration of all the results, this contributes to the fact that statements on the benefits of CBT or GET were classified in the lowest level of certainty (“hint”). (In German, “Verblindung”/“fehlende”“Verblindung” ist in Nutzenbewertungen des Instituts immer ein Aspekt bei der Einschätzung des Verzerrungspotenzials. In diesem Bericht wurde zum Beispiel der Studie PACE ein hohes Verzerrungspotenzial attestiert. Dies trägt in der endpunktübergreifenden Gesamtabwägung aller Ergebnisse dazu bei, dass Aussagen zum Nutzen der CBT bzw. GET in die niedrigste Stufe der Aussagesicherheit eingeordnet wurden (“Anhaltspunkt”) (p. 156). However, despite the earlier mentioned recommendation in the IQWiG methods guidance handbook not to rely on subjective outcomes in non-blinded studies but to use objective ones instead, they ignored their own recommendation.

Moreover, another problem with outcomes assessed via questionnaires in a non-blinded trial is “response-shift bias”. This occurs when an intervention leads individuals to change their evaluation standard regarding the dimension measured, leading the therapist (and, often, also the patient) to conclude erroneously that the treatment has worked [29,33]. This form of bias is even more of a problem in CBT trials for ME/CFS, because this specific form of CBT aims to modify participants’ beliefs and perceptions of their symptoms [34]. Consequently, a better score after treatment has ended can simply reflect answering a questionnaire in a different way than at baseline. The only way to correct for that is by using objective outcomes.

Further bias was introduced into those studies because of “a strong allegiance towards the therapy, and anything that increases expectations and hope in participants” (p. 428 [35]). Part of the issue of allegiance bias in psychological therapies is that measures are often subjective, and the clinician may unconsciously prod the subject to respond to their favored therapy or not respond to a therapy they consider ineffective. For example, a therapist’s confidence in their therapy of choice is almost certainly perceptible to the patient, even when this is not overtly advertised [36]. Generally, in studies examining more than one treatment approach, the treatment favored by the researchers tends to outperform other treatments [37,38,39]. Several factors may contribute to this effect, but one is likely to be the way the non-favored, comparison treatment is conceptualized and implemented. Often, when a treatment is used as a comparison condition, it may be implemented in a weaker form then when it is used clinically. Usually, investigators may not believe in the effectiveness of the control condition and are less enthusiastic in their delivery of that treatment, which, in turn, may influence the outcome [23,40]. Consequently, treatments might not be presented to participants as equally likely to lead to improvement. This is especially important when the study is non-blinded, and the primary outcomes are self-reported measures that can be strongly influenced by patients’ expectations. Finally, a researcher’s enthusiasm for a particular treatment can also lead them to over interpret their findings or overlook limitations [40]. 

Before they conducted their research, psychiatrist Professor White (principal investigator of the PACE trial and the GETSET trial [20,32]) and psychologist Professor Bleijenberg, an author of Janse et al. [24], were known to favor the approach to the illness being tested. These investigators are strong proponents of the ‘unhelpful cognitions’ theory of ME/CFS, which they and other colleagues originated and/or actively promoted. If their trials had failed to show significant improvement and recovery through CBT and/or GET, this would have undermined the very theories of reversibility to which the investigators have dedicated their careers. Consequently, the risk of latent bias was palpable from the outset [41,42]. 

During the non-blinded PACE trial that relied on two subjective primary outcomes, the bias was increased further, because patients were sent a newsletter that stressed that, in the NICE guidelines from 2007, “recommended therapies include Cognitive Behavioural Therapy, Graded Exercise Therapy and Activity Management” [43].

Finally, why the report did not use or analyze all the objective outcomes that were used by the studies, in view of all these forms of bias, is unclear. Even more so because the authors of the GETSET trial themselves state the following about that. “All outcomes were self-rated, which might lead to bias by expectation…We did not measure any objective outcomes, such as actigraphy, which might have tested the validity of our self-rated measures of physical activity” p. 372) [30]. Consequently, the GETSET authors themselves are not sure if their exercise therapy led to real improvement or not, because they did not use objective outcomes. 

### 3.4. Selection of Patients Who Do Not Have the Disease under Investigation

As mentioned above, according to the IQWiG report, the presence of PEM is a central diagnostic criterion [1]. The IQWiG used the NICE ME/CFS guidelines from 2021 and its evidence reviews of the effectiveness of non-drug and drug-based interventions for patients with ME/CFS to select the studies for its report. (In German: “Für die Evidenzkartierung wurden die vom NICE erstellten Evidenzreviews der Wirksamkeit nicht medikamentöser und medikamentöser Interventionen für Patientinnen und Patienten mit ME/CFS herangezogen” p. 29). It notes that NICE did not use studies in which less than 95% of a study’s population suffered from PEM. However, the IQWiG then used studies from that guideline whereby at least 80% of the study population were suffering from PEM without explaining why it changed 95% or more into 80% or more. (“Im vorliegenden Bericht wurden für die Ergebnisdarstellung in der Evidenzkartierung diejenigen Studien herangezogen, bei denen der in NICE 2021 berichtete PEM-Anteil der Population mindestens 80% beträgt” p. 30). It also did not explain why it is acceptable for an evidence review to include trials where up to 20% of patients (or, to put it differently, up to one in five of every patient) did not have the disease under investigation. However, it was likely to be more than that, because the PACE trial used the Oxford criteria (six months of chronic disabling fatigue). PEM is not necessary for a diagnosis, according to these criteria [44]. The GETSET study used the “NICE criteria [from 2007 which] require at least 4 months of clinically evaluated, unexplained, persistent, or relapsing fatigue with a definite onset that has resulted in a substantial reduction in activity and that is characterised by postexertional malaise or fatigue, or both. They also require at least one of ten related symptoms…” p. 364). However, post-exertional fatigue is not a characteristic of the disease but is simply a normal physiological response to exercise. Moreover, these criteria have been dropped by NICE in their updated guidelines from 2021, because they were too lax and led to the inclusion of a substantial number of patients who do not have ME/CFS. 

Janse et al. [24] used the Fukuda criteria. PEM is optional according to these criteria. Most (97%) (155/160) of the participants in the treatment groups in this study fulfilled these criteria, yet only 89% of the participants in both treatment groups had PEM. The PACE trial reported that 84% (CBT) and 82% (GET) were suffering from PEM, and the GETSET study reported that it was 100%. Yet, according to the final version of the PACE trial protocol, PEM was defined as “feeling ill after exertion” (p. 156) [45]. According to the IQWiG report, PEM is characterized by a decrease in functioning and a worsening of other ME/CFS symptoms after physical or cognitive exertion and typically occurs with a time lag (hours or days) after physical or mental activity or stress. (In German, “Sie ist durch eine Abnahme des Funktionsniveaus und eine Verschlechterung weiterer ME/CFS-Symptome nach körperlicher oder kognitiver Anstrengung gekennzeichnet und beginnt typischerweise erst zeitversetzt (Stunden oder Tage später) nach einer körperlichen oder geistigen Aktivität oder Belastung” p. 27,28). Consequently, PEM in the PACE trial was something else. This is acknowledged by the report when they write the following: “These data related to the proportion of patients with (at least) one reported PEM event in the previous week (see Table 46 [of the report]). Caused by the non-specific question about “feeling ill after exertion” and the assumption that the question was mostly answered by the patients themselves without further explanation, *it can be assumed that these results do not only include actual PEM events in the sense of a delayed worsening of most of the symptoms* [italics by us]. Rather, it can be assumed that it includes all post-activity overload events, regardless of the severity and duration of the activity-related illness. This interpretation is supported by the fact that a very high proportion of patients, 49% (CBT) and 63% (SMC), recorded a corresponding event in just 1 week. In view of the severity of an actual PEM event, however, it seems very unlikely that around half of the participants experienced PEM in the week surveyed.” (In German, “Diese Daten bezogen sich auf den Anteil der Patientinnen und Patienten mit (mindestens) einem berichteten PEM-Ereignis in der vorausgegangenen Woche (siehe Tabelle 46). Bedingt durch die unspezifische Fragestellung nach Krankheitsgefühl nach Anstrengung (“feeling ill after exertion” und die Annahme, dass die Frage von den Patientinnen und Patienten zumeist selbst ohne weiterführende Erläuterung beantwortet wurde, ist davon auszugehen, dass diese Ergebnisse nicht nur tatsächliche PEM-Ereignisse im Sinne einer zeitlich verzögerten Verschlechterung der Mehrheit der Symptome umfassen. Vielmehr ist davon auszugehen, dass darin jegliche Überlastungsereignisse nach Aktivität beinhaltet sind, unabhängig von der Schwere und Dauer des aktivitätsbedingten Krankheitsgefühls. Diese Interpretation wird dadurch gestützt, dass mit 49% (CBT) bzw. 63% (SMC) ein sehr hoher Anteil der Patientinnen und Patienten in nur 1 Woche ein entsprechendes Ereignis protokollierten. Vor dem Hintergrund der Schwere eines tatsächlichen PEM-Ereignisses erscheint es jedoch sehr unwahrscheinlich, dass bei rund der Hälfte der teilnehmenden Personen in der abgefragten Woche eine PEM auftrat” p. 139). It is unclear why the report ignored this and did not conclude that the percentage of participants with PEM in the PACE trial (84% CBT; 82% GET) was therefore likely to be substantially lower than claimed by the study. The Fukuda criteria [46], were the only criteria used by Janse et al. [24] but were also used by the other two studies. As PEM is optional for a diagnosis and not a requirement, it would be logical that the percentage of participants with PEM is lower than the percentage of participants diagnosed with ME/CFS according to these criteria. That is also exactly what was found by Janse et al. [24]. If we use the ratio of 92%, the percentage of participants diagnosed with ME/CFS according to the Fukuda criteria who were suffering from PEM from Janse et al. [24] (89/97 = 92%), and apply that to the percentage of participants diagnosed with ME/CFS according to the Fukuda criteria in the other two studies, then we get a more realistic percentage of participants in those studies who suffered from PEM as defined by the IQWiG report. As can be seen in Table 1, the percentage of participants suffering from PEM in the PACE trial and the GETSET study was 62% (CBT) and 61% (GET) in the PACE trial and 68% (GES) in the GETSET study. Consequently, the report should have excluded the PACE trial and the GETSET study from their analysis, because the percentage of participants in both treatment groups was lower than the 80% or more that was required according to the report to be included in their analysis. 

It also suggests that a substantial number of patients in the PACE trial and the GETSET study did not have the disease under investigation. Participants who do not suffer from the disease under investigation should be excluded from a properly conducted study, because “errors in the diagnosis of a condition” result “in erroneous inferences of improvement” in its absence (p. 363 [29]). Even more so because these participants did not suffer from PEM or exertion intolerance, contrary to ME/CFS patients. This is an even bigger problem in a study that was testing the efficacy of an exercise treatment (GET) and a psychological treatment with an exercise component (CBT for ME/CFS). Why the PACE trial and the GETSET study did not exclude these patients is unclear, nor is it clear why the report did not exclude these studies. 

### 3.5. Problems with the Chalder Fatigue Scale

All three studies used the Chalder Fatigue Scale, even though there are many issues with it, as found by the reanalysis of the Cochrane CBT review [7]. Some of its main problems are the ceiling effect and the fact that it has two official ways of scoring, yet, if you change from one official way of scoring to the other, then you can get a different result, as was highlighted by the FINE trial (Fatigue Intervention by Nurses Evaluation trial) [47], the sister trial of the PACE trial. The FINE trial reported a null effect in their original publication in 2010, one year before the PACE trial was published, using bimodal scoring. Yet, when they changed their scoring in their mediation article from bimodal to Likert scoring, they were able to report a small but statistically significant improvement [48]. This is of particular importance and concern, because the PACE trial also changed their way of scoring from bimodal to Likert, and it might well be that this also changed a null effect into a positive effect. Of note is that Alison Wearden, the principal investigator of the FINE trial, was a member of the PACE trial Steering Committee [20]. 

Another major problem of the Chalder Fatigue Scale is the ceiling effect, which refers to the fact that, if patients have the maximum score at the baseline, if they deteriorate, then that is not reflected in their scores. In the PACE trial, 44% of participants had a Likert score of 30–33 at baseline (scale from 0 to 33, higher scores indicating more fatigue) [15]. If, for example, a participant with a score of 33 deteriorated for example in eight items but improved in three, then they would not have been classified as deteriorated by five but as improved by three. Consequently, the Chalder Fatigue Scale is an unreliable outcome measure. The unreliability of this instrument and the fact that it can lead to false-positive outcomes was also demonstrated by a 2003 study by Tench et al. on the efficacy of exercise therapy for systemic lupus erythematosus (SLE) [49]. Tench et al. included Professor White, the principal investigator of the PACE trial and the GETSET trial, and they compared the efficacy of 12 weeks of graded exercise therapy with two other interventions (relaxation and no intervention). We mention this, because the Chalder Fatigue Scores after GET improved by three points compared to relaxation and by four compared to the no-treatment control group. This is similar to the improvements in the PACE trial [20], which, according to the IQWiG report [1], provided evidence of the efficacy of CBT and GET for mild and moderate ME/CFS. However, GET did not lead to improvement on the other two fatigue scales used by Tench et al. (the Fatigue Severity Score (FSS) and the Visual Analogue Scale (VAS)), nor did it lead to an objective improvement (VO2 peak) as found by a review [15].

### 3.6. Analysis of the Three Studies Used by IQWIG

#### 3.6.1. Janse et al. [24]

This study used the Fukuda criteria, yet “six patients were included with less than four CDC symptoms”, and five of those were from the two treatment groups (p. 114). Additionally, “twenty-five patients started another treatment for CFS during the study” (p. 114) but were not excluded from the study. Even though patients who do not have the disease under investigation should be excluded from a properly conducted trial, the same applies to patients who are starting a different treatment during a trial, because then, it becomes impossible to know if any improvement is down to the treatment under investigation or to the new treatment or a combination of the two. By not excluding these patients, the researchers introduced unnecessary bias into their study. The percentages of paid jobs of 65% (protocol-driven CBT or pdCBT) and 71% (on-demand CBT or odCBT) and the physical functioning scores of 62.4 (pdCBT) and 62.9 (odCBT) at the baseline suggest that patients were only mildly affected. Moreover, with these physical functioning scores, patients would have already been classified as recovered in the PACE trial, for which a score of 60 or more was needed. Additionally, the following figures suggest that the control group was not properly matched and that patients in this group were more disabled than in the other two groups, which would have biased the study further. Duration of complaints in years: 4 (pdCBT), 4.5 (odCBT) and 6.5 (waiting list or WL); overall impairment: 1452 (pdCBT), 1496 (odCBT) and 1608 (WL); clinically relevant depressive symptoms: 31% (pdCBT) and 29% (odCBT) and 41% (WL) and multiple joint pain: 71% (pdCBT), 73% (odCBT) and 80% (WL). Other problems with this study are discussed elsewhere in our analysis.

#### 3.6.2. The GETSET Trial [30]

At baseline, patients had been ill for 46 (23–114, GES) and 42 months (25–99, SMC), and their level of physical activity was 120 (30–360, GES) and 185 min per week (75–570, SMC). These figures suggest that many patients had been ill for less than three years, and improvement or recovery might simply be spontaneous, as, according to an extensive review, the chances of spontaneous recovery are the highest for patients who have been ill for less than two to three years [8]. Additionally, judging by their level of physical activity, these patients were only very mildly affected at best. 

According to the study itself, “five patients would need to be treated for one to benefit from GES” (p. 370). Yet, the participants’ rated positive change in CGI of their illness from the baseline was 14% (GES) and 6% (SMC). Consequently, only 8% benefited subjectively from GES. Therefore, the authors should have concluded that 12 patients would need to be treated for one to benefit subjectively from GES. This would also mean that 11 patients would receive the treatment without any subjective benefit. 

Moreover, according to the IQWiG report [1], the patients in the control group had access to the self-help brochure on how to carry out the GET independently 12 weeks after randomization. Additional support or guidance from trained therapists, as in the intervention group, was not described. It is therefore assumed that these people were still suitable as a control group and that it would make sense to look at the long-term data. (In German, “Die Patientinnen und Patienten der Kontrollgruppe hatten erst nach 12 Wochen nach Randomisierung Zugriff auf die Selbsthilfe-Broschüre zur selbstständigen Durchführung der GET. Eine ergänzende Unterstützung oder Anleitung durch geschulte Therapeutinnen und Therapeuten wie in der Interventionsgruppe wird nicht beschrieben. Daher wird davon ausgegangen, dass diese Personen weiterhin als Kontrollgruppe geeignet sind und eine Betrachtung der Langzeitdaten sinnvoll ist” (p. 82)). Yet, from that moment on, they crossed over to the treatment group and were treated with the treatment under investigation. The consequence of this is that, from that moment onwards, GETSET became a non-controlled study, i.e., a study without a control group. Due to that, from then on, it’s impossible to know if any improvements are down to the treatment under investigation or the absence of a control group. Other problems with this study are discussed elsewhere in our analysis.

#### 3.6.3. The PACE Trial [20]

This non-blinded study, which is the biggest CBT trial ever conducted (*n* = 641), used two subjective primary outcomes: fatigue and physical functioning. Its protocol was published two years after the study started [45,50,51], even though a protocol should be published before the start of a study, because, as noted by Evans, “a fundamental principle in the design of randomized trials involves setting out in advance the endpoints that will be assessed in the trial, as failure to prespecify endpoints can introduce bias into a trial and creates opportunities for manipulation” (p. 0001 [52]).

The trial also made an extensive number of endpoint changes that created an overlap in the entry and recovery criteria. For example, the physical functioning score needed to be classed as recovered was changed from 85 or more to 60 or more. At the same time, the entry score was changed from 60 or less to 65 or less. Consequently, with a physical functioning score of 60 or more but not more than 65, one was simultaneously ill and recovered at the same time. Similar changes were made to the entry and recovery scores of the Chalder Fatigue Scale so that, with a Chalder Fatigue Score of 18, one was simultaneously ill and recovered at the same time [51]. Something such as that should not happen in a properly conducted and properly peer reviewed trial. 

Moreover, “Queen Mary University of London, which oversaw the trial,…spent almost £250,000 of public money on legal fees” trying to prevent the release of individual participant data, yet “they lost” (p. 1190 [53]). Wicherts et al. [54] explored “authors’ reluctance to share data” in psychological studies and found that the “willingness to share research data is related to the strength of the evidence and the quality of reporting of statistical results” (p. 1, 4). Wicherts et al. concluded that it was “rather disconcerting that roughly 50% of published papers in psychology contain reporting errors and that the unwillingness to share data was most pronounced when the errors concerned statistical significance” (p. 5 [54]). Those individual data were finally released [18,19], as ordered by a judge after a patient won his case against the PACE trial under the British Freedom of Information Act [55]. When these data were analyzed, it became clear that they did not back up the claims of the authors [6,56]. If the PACE trial would have stuck to the endpoints as defined in the protocol, then there was no statistically significant difference between the results of all the four groups, as found by Wilshire et al. [6]. 

Additionally, according to the baseline quality-of-life scores, the groups were not evenly matched, and patients in the GET (0.52) and CBT (0.54) groups were less disabled than in the APT group (0.48) [57]. 

After treatment was deemed to be successful, the fatigue scores at 52 weeks were 20.3 (CBT) and 20.6 (GET) and physical functioning scores 58.2 (CBT) and 57.7 (GET). Yet, the entry scores for fatigue and physical functioning were 18 or less and 65 or less, respectively [20]. On top of that, a physical functioning score of 70 represents significant reductions in physical functioning [58], and a score of 65 or less represents “abnormal levels of physical function” (p. 2229), according to the PACE trial itself [59], and severe disability, according to the literature [60]. Moreover, according to a review of the Chalder Fatigue Questionnaire by Jackson [61], a “binary [bimodal] fatigue score of 3 or less represents…not fatigued, with scores of 4 or more equating to ‘severe fatigue’” (p. 86). The PACE trial’s original bimodal fatigue recovery criterion was 3 or less [20], and a bimodal fatigue score of 3 equates to a Likert score of 6–9 and a bimodal score of 4 to a Likert score of 8–12. Consequently, many patients were still ill enough to reenter the PACE trial to be treated with the same “effective” treatments again. This result is even worse if one realizes that 13.3% of participants were already classed as recovered according to one or two of those scores at trial entry, before they received any treatment [56]. Additionally, after “effective” treatment, most patients were still severely disabled, according to the fatigue and physical functioning scores. Such a treatment cannot be deemed to be effective. Other problems with this study are discussed elsewhere in our analysis.

### 3.7. Depression

A group of international ME/CFS experts [62] concluded in 2003 that “the presence of a medical or psychiatric condition that may explain the chronic fatigue state excludes the classification as CFS in research studies because overlapping pathophysiology may confound findings specific to CFS” (p. 6). This study included two leading CBT researchers from the UK and the Netherlands, psychiatrist Professor White, who was the principal investigator of the PACE trial and the GETSET trial, and psychologist Professor Bleijenberg, who was part of Janse et al. In that study, 31% (pdCBT) and 29% (odCBT) in the two treatment groups suffered from clinically relevant depressive symptoms. In the PACE trial, it was 32% (CBT) and 34% (GET), and 9% in the treatment group in the GETSET trial. Why those studies did not exclude these patients is unclear. Even more so because it is well known that CBT is the most effective treatment for depression [51]. Consequently, any improvement in those groups might simply be down to an improvement of depressive symptoms.

### 3.8. Quality of Life and CFS Symptom Count Scores

According to the IQWiG report, no results could be identified in the PACE trial for the European Quality-of-Life 5 Dimensions Questionnaire (EQ-5D). (In German: “Auch in der Studie PACE konnten keine Ergebnisse zur geplanten Erhebung des European Quality of Life 5 Dimensions-Fragebogen (EQ-5D) identifiziert werden” (p. 141)). It is easy to miss those results, because they were not published in the original PACE trial paper in 2011 [20]. Instead, the authors published those in their cost-effectiveness analysis [57]. The net improvement of the quality-of-life scores (EQ-5D) in the PACE trial at the end of treatment was minimal (2.3% after GET over APT and 0.5% after CBT over APT) [5]. This is even though, according to the baseline quality-of-life scores, the groups were not evenly matched, and patients in the GET (0.52) and CBT (0.54) groups were less disabled than in the APT group (0.48). Additionally, the quality-of-life scores after treatment with GET (0.60) and CBT (0.61) were similar to the score (0.60) for people with five or more chronic health conditions and were still worse than in cerebral thrombosis (0.62), rheumatoid arthritis and angina (0.65), acute myocardial infarction (AMI) (0.66), MS (0.67), lung cancer (0.69), stroke (0.71) or ischemic heart disease (0.72) (higher scores indicating a better quality of life) [63,64]. 

According to the IQWiG report [1], Janse et al. did not report their quality-of-life scores. (In German, “In der Janse 2018 wurden Ergebnisse zum Endpunkt gesundheitsbezogene Lebensqualität (HRQoL) trotz geplanter Erhebung mittels European Quality of Life 6 Dimensions Fragebogen (EQ-6D) nicht berichtet” (p. 141)). This is known as selective outcome reporting bias, which can potentially compromise the validity of a trial and usually happens when those results contradict the conclusions of a study [29,40,65].

The chronic fatigue syndrome symptom count is another relevant patient-reported outcome, as the cognitive behavioral model (CB model) claims that CBT and GET lead to recovery. The only trial that used this outcome is the PACE trial [20]. Figures for the end of treatment were not released, but there was no statistically significant difference in the improvement in the chronic fatigue syndrome symptom count at 52 weeks between GET and SMC (*p* = 0.0916), GET and APT (*p* = 0.23) or between CBT and APT (*p* = 0.0986) [57]. 

### 3.9. Analysis of the Objective Outcomes

As noted, the IQWiG report [1] reported a null effect on the six-minute walk test in the PACE trial but then ignored this in the rest of the report. Moreover, the report did not use or analyze the other objective outcomes used by the studies, even though, for example, Janse et al. [24] acknowledged the ineffectiveness of their therapy in an indirect way when they wrote the following. “A *post hoc* analysis showed that objectively assessed physical activity significantly increased after iCBT. However, this might be an accidental finding, taking the amount of missing data into account and previous research that did not find an increase in physical activity following CBT” (p. 116). This also signals a problem with a high number of dropouts in that study, which might be an indication that the treatment was not effective and not safe either. 

The GETSET trial [30] did not use objective outcomes. The authors themselves noted that “we did not measure any objective outcomes, such as actigraphy, which might have tested the validity of our self-rated measures of physical activity” (p. 372). Consequently, the GETSET authors themselves are not sure if graded exercise therapy led to a real improvement or not. 

The PACE trial [20] used the actometer at the beginning of the study but not at the end, because they deemed it too much of a burden for patients [51], even though they concluded that 22% had recovered and 60% had improved in the CBT and GET groups. Consequently, it should have been easier at the end of treatment than before treatment started. Their step test did not show any objective improvement. The report discusses the results of the six-minute walk test in the PACE trial in Section 5.6.6 (“Ergebnisse zur körperlichen Leistungsfähigkeit” p. 116–119). It concluded that CBT and GET did not lead to any objective short, medium, or long-term improvement, nor did the six-minute walk tests show that CBT and GET were harmful. (In German, “Insgesamt konnte für den Vergleich CBT versus SMC für keinen der Auswertungszeiträume (kurz-, mittel- oder längerfristig) ein Anhaltspunkt für einen Nutzen oder Schaden der CBT im Vergleich zur SMC hinsichtlich des Endpunkts körperliche Leistungsfähigkeit abgeleitet werden” p. 116–117, and “Insgesamt ergibt sich für den Vergleich GET versus SMC für den Endpunkt körperliche Leistungsfähigkeit für keinen der Auswertungszeitpunkte (kurz-, mittel- oder längerfristig) ein Anhaltspunkt für einen Nutzen oder Schaden der GET im Vergleich zur SMC” p. 119). Moreover, even in the GET group, where participants exercised five times a week for 26 weeks, they were not able to walk more than 379 m. In comparison, the normal distance walked for women of a similar age of 38 (mean age in the PACE trial) is 600 m or more and 659 m or more for men. The individual released 6MWT data showed that only two people in the trial (one in the CBT and one in the APT group) achieved more than 600 m but less than 659 [18,19,56]. As sex was removed from these data, it is unclear if these patients reached the normal levels or not. 

It is unclear why the report [1] states that research suggests that cognitive behavioral therapy can… help people [with ME/CFS] return to school or work earlier. (In German: “Die Forschung deutet darauf hin, dass eine kognitive Verhaltenstherapie etwas bewirken kann. So konnte sie in Studien dazu beitragen, die Erschöpfung bei ME/CFS zu lindern und dabei helfen, früher in die Schule oder zum Arbeitsplatz zurückzukehren” IQWiG Gesundheitsinformation Entwurf Mehr Wissen: ME/CFS (p. 288 of the pdf), as the PACE trial showed that CBT had no effect and GET had a negative effect on employment status. Additionally, more patients were reliant on illness and disability benefits after treatment with CBT and GET than before treatment with it [8,57]. On top of that, an extensive review of the effect of CBT and GET on the employment and disability benefits status concluded that “our review shows that more patients are unable to work after treatment than before treatment with CBT and GET” (p. 1 [66]). Why the IQWiG report ignored this and the PACE trial’s null effect on the step test and occupational and benefits status in their conclusion is unclear.

### 3.10. Adherence to Therapy

A treatment can only be said to be effective if patients adhere to it. In Janse et al. [24], “a substantial number of patients did not fully adhere to the interventions” (p. 117). According to the figures published by the trial itself, only 16% (odCBT) and 19% (pdCBT) of participants in the treatment groups adhered fully to the interventions. This also means that *a substantial number of patients* who did not adhere to the interventions equates to 84% (odCBT) and 81% (odCBT) of participants in the treatment groups. In the GETSET trial [30], “the physiotherapists reported that 43 participants (42%) adhered to GES completely or very well, 31 (30%) moderately well, and 30 (29%) slightly or not at all” (p. 370). The figures presented by the physiotherapists suggest that, in up to 59% of cases, the treatment was not very well tolerated by even mildly affected patients. 

The PACE trial [20] defined adequately treated, as when patients had followed 10 or more treatment sessions out of 16. According to their figures, 87% (CBT) and 85% (GET) were adequately treated. However, 10 out of 16 sessions equates to an attendance of 62.5% of the sessions. If 10 sessions would be adequate, then it would be wasting money and time to set up a trial of 16 sessions. An attendance of 62.5% would be labeled as bad attending and wasting valuable therapy time in real life. In a more recent study by Smakowski et al. [67] on the efficacy of GET in a London CFS clinic that involved Professor Chalder, one of the three principal investigators of the PACE trial, an adequate number of treatment sessions was 12, which would equate to 75% in the PACE trial. The PACE trial does not provide numbers on how many patients followed 12 sessions, but it is likely to be less than 87 and 85%; otherwise, it is likely that the PACE trial would have used 12 sessions instead of 10. 

As noted by Heneghan et al. [68] in their article entitled “Why clinical trial outcomes fail to translate into benefits for patients”, there is the ‘5 and 20 rule’. According to this rule, if the percentage of dropouts, missing data or patients who do not adhere to the treatment is more than 20%, then the study is highly biased; if it is less than 5%, then this represents a low risk of bias. Moreover, according to the study by Lilienfeld et al., “Why Ineffective Psychotherapies Appear to Work: A Taxonomy of Causes of Spurious Therapeutic Effectiveness” [29], patients who drop out or do not adhere to treatment are usually the ones who do not benefit from the treatment or are negatively affected by it. Consequently, treatments with such low adherence rates, i.e., CBT and GET, as mentioned above, cannot be described as safe and effective. The Supplementary Materials of the study by Smakowski et al. [67] showed that, in real life, outside the confines of a clinical trial, 40% (37/95) of patients attended less than the standard number of treatment sessions, and attendance for 11% of patients was unaccounted for. It would be reasonable to assume that data were missing for these patients, because they dropped out of treatment before assessment was complete. Thus, less than half of patients adhered to the treatment and completed the treatment program. Such poor adherence indicates that GET is poorly tolerated and unacceptable to even mildly to moderately affected patients [69]. Despite the many problems of the study by Smakowski et al. [67], it showed that the patients remained severely disabled after treatment with GET in real life [69]. Something similar was found by an analysis of the efficacy of CBT and GET in 11 CFS clinics in the UK by Collins and Crawley [70]. Treatment with GET in real life by Dutch sports physicians not only showed that the occupational status did not improve, but it also showed very high dropout rates after 6, 9 and 12 months of 55%, 73% and 80% [5,71]. In view of such bad adherence to the treatment, one cannot conclude that CBT and GET are safe and effective treatments.

Moreover, the IQWiG methods guidance handbook states that, as a rule, results are not included in a benefit assessment if they are based on less than 70% of the study participants, i.e., if the proportion of study participants who are not included in the analysis is greater than 30%. In the literature, some reviews do not consider studies to be meaningful if it is more than 20%. (In German, “Ergebnisse fließen in der Regel nicht in die Nutzenbewertung ein, wenn diese auf weniger als 70% der in die Auswertung einzuschließenden Studienteilnehmer basieren, das heißt, wenn der Anteil der Studienteilnehmer, die gar nicht in der Auswertung berücksichtigt werden, größer als 30% ist. In der Literatur werden zum Teil bereits Auswertungen, in denen 20% der Studienteilnehmer nicht berücksichtigt werden, als nicht mehr aussagekräftig betrachtet” p. 198). The proportion of participants who did not adhere to the treatment of up to 59% in the GETSET trial [30] and 84% (odCBT) and 81% (odCBT) of participants in Janse et al. [24] is substantially more than 20 or 30%. Consequently, both studies should have been excluded from the analysis. 

### 3.11. Safety of GET and CBT for ME/CFS

The IQWIG [1] writes the following about the safety of CBT and GET. In all three included studies, information on possible damage, such as induced PEM, was missing. However, as there was a tendency towards an improvement on four subjective endpoints (fatigue, sleep quality, physical function and mental status), it can be assumed that both treatments are not harmful. (In German, “Fehlende Angaben zu möglichen Schäden wie induzierter PEM. In allen 3 eingeschlossenen Studien ist unklar, inwieweit die Häufigkeit spezifischer Schadensereignisse wie bspw. der wesentliche Schadensaspekt wiederholter PEM-Ereignisse (“Crash”), induziert durch die interventionsbedingte kognitive, emotionale oder körperliche Anstrengung, erfasst wurde. Da jedoch die Ergebnisse zu den Endpunkten Fatigue, Schlafqualität, körperliche Funktion und psychischer Status in Richtung eines Vorteils für die beiden Interventionen tendieren, ist nicht davon auszugehen, dass sich mögliche nicht dokumentierte ME/CFS-spezifische Schadensereignisse wesentlich auf diese Endpunkte ausgewirkt haben und die Richtung der beobachteten Ergebnisse damit in Gänze infrage gestellt werden können. Hätten sich ME/CFS-spezifische Schadensereignisse, insbesondere PEM, aufgrund der Intervention gehäuft gezeigt, ist anzunehmen, dass sich dies auch negativ auf die beobachteten Ergebnisse ausgewirkt hätte und sich keine Vorteile bspw. bei dem Endpunkt Fatigue hätten erkennen lassen können” p. 152).

However, there are several problems with this assumption. If there is no information about possible harm, then that does not mean to say that a treatment is safe. All it means is that these studies provided no evidence that CBT and GET are safe. On top of that, if we would, for example, take the evidence mentioned below about the harmfulness of CBT and GET into account, then it is much more likely that the tendency towards an improvement on four subjective endpoints was only a tendency towards an improvement instead of a real improvement, or even total remission of symptoms, because so many patients were negatively affected by these treatments, and only the patients who did not have the disease under investigation and were not excluded from the studies benefited subjectively from the treatments. Moreover, the safety of patients should always come first, and the *Do No Harm principle* is the most important principle of medicine [72]. If a study cannot guarantee the safety of a particular treatment, then that treatment should not be recommended or used.

Downing et al. [73] investigated the post-market safety of 222 novel therapeutics after the initial regulatory approval. They found that “there were 123 new post market safety events (3 withdrawals, 61 boxed warnings, and 59 safety communications) during a median follow-up period of 11.7 years…affecting 71 (32.0%) of the novel therapeutics” (p. 1854). They also concluded that “biologics, *psychiatric therapeutics [italic by us]*, and accelerated and near–regulatory deadline approval were statistically significantly associated with higher rates of events” (p. 1854).

CBT for ME/CFS contains an element of GET, and a key feature of GET is pushing beyond limits. In the PACE trial’s GET manual for participants, participants were told to interpret symptom flares as “a normal part of CFS/ME recovery” and not as a worsening of the disease and these symptom flares “are likely to become less severe and last for less time than previously as I get stronger” (p. 81 [74]). In the patient booklet of the FINE trial [75], which was the sister trial of the PACE trial, patients were told that “activity or exercise cannot harm you” and that “medical research evidence shows…no underlying serious disease” (p. 37, 49) but, also, that “you will have conquered CFS by your own effort and you will be back in control of your body again” (p. 39). That booklet also states that “you cannot relapse because you now know how to combat it” (p. 93). However, as noted by the IQWiG report [1], symptom exacerbation after mild physical or mental activity exertion, which usually starts hours or days later, is called post-exertional malaise (PEM) and is a central diagnostic criterion. The duration of PEM is unpredictable and can last for hours, days and weeks. (In German, “heute ist das Vorliegen einer PEM ein zentrales diagnostisches Kriterium” p. 1) [76]. “Typisch bei ME/CFS ist, dass sich die Symptome häufig schon nach leichten körperlichen oder geistigen Aktivitäten verschlimmern und dann tage- oder wochenlang anhalten können” p. 255 [1]). ME/CFS ist gekennzeichnet „durch das Leitsymptom der Symptomverschlimmerung nach Anstrengung (Post-exertional Malaise (PEM))” p. 159) und “die Schwere und Dauer der Symptomverschlimmerung sind dabei unabhängig vom Auslöser. Eine PEM beginnt typischerweise erst Stunden oder Tage nach einem Auslöser, manchmal auch unmittelbar danach. Die Dauer einer PEM ist nicht vorhersehbar und kann Stunden, Tage und Wochen andauern” (p. 6 [1]).

The consequence of using CBT and GET as described above is that patients are forced to go over their limits, because the cognitive behavioral model assumes that they are caused by deconditioning and not by an underlying disease. Yet, when patients go over their limits, then patients get symptom exacerbations and relapses, as noted by the IQWiG report, and the bigger those exacerbations and relapses are, the less likely it is that patients will recover from them. 

Patient surveys have highlighted the harmfulness of CBT, which contains an element of GET and GET for more than 20 years. Kindlon [77] and Geraghty et al. [78], who pooled patients’ surveys (*n* = 1808, 5 surveys and *n* = 3251, 10 surveys in 2011 and 2017, respectively), found that 20 percent of respondents reported that CBT had worsened their health, and for GET, this was at least in 50% of cases. In a 2 to 5-year follow-up after treatment in NHS CFS clinics with CBT and GET, 13.5% were ‘a little worse’ and 17.1% were ‘worse’ or ‘very much worse’ [70]. The British National Institute for Health and Clinical Excellence (NICE) published its updated ME/CFS guidelines in October 2021 [4]. As part of that review process, it commissioned the Oxford Brookes University to carry out a survey amongst ME/CFS patients (*n* = 2274) on the safety of CBT and GET. When they published their report in February 2019 [79], they found the following: 98.5% of the patients who took part in the survey experienced post-exertional malaise, the core symptom of the disease. Worsening of the symptoms after treatment was reported by

- 81.1% (GET);
- 58.3% (CBT).

After treatment, the percentage of severely affected patients increased from

- 12.9% to 35.3% (GET);
- 12.6% to 26.6% (CBT).

The very high number of symptom flareups and relapses is not surprising, as PEM is the main characteristic of ME/CFS. Studies by, for example, Black and McCully [80] provided objective evidence for this. They concluded “that CFS patients may develop exercise intolerance...after 4–10 days. The inability to sustain target activity levels, associated with pronounced worsening of symptomology, suggests the subjects with CFS had reached their activity limit” (p. 1). Lien et al. [81] found that exercise deteriorates the physical performance and increases lactate in patients with ME/CFS, whereas, in the healthy population, the exact opposite happens. Kujawski et al. [82] specifically looked at the effects of exercise in ME/CFS. They concluded that “exercise was not well tolerated by 51% of patients” (p. 1). Moreover, many studies have provided objective evidence for exertional intolerance, delayed muscle recovery and other physical abnormalities in ME/CFS following exercise, which were not seen in the healthy (sedentary) controls [83,84,85,86,87,88,89,90,91,92].

Finally, the very high dropout rates after 6, 9 and 12 months of 55%, 73% and 80%, respectively, as found by an analysis of the efficacy of GET in the sports medical department of a Dutch hospital [5,71], highlight the inappropriateness of this treatment, even in only very mildly affected patients and even when it is tailormade to the patient and done at 50 to 60% of the VO2max.

Given all these considerations, one cannot conclude that CBT and GET are safe. The safety of patients should always come first, and if one does not know if a treatment is safe or, as in this case, there is ample evidence that both treatments are harmful, then those treatments should not be used, promoted or recommended.

### 3.12. Excluded Study

The study by Núñez et al. [93] is not mentioned in the IQWiG report [1]. This trial compared multidisciplinary treatments combining CBT, GET and pharmacological treatment with the usual treatment. It found that, at twelve months follow-up, the interventions did not improve health-related quality-of-life scores and led to worse physical function and bodily pain scores. Núñez et al. concluded that “the results of our study tend to support the somewhat controversial findings of Twisk and Maes that the combination of CBT and GET is ineffective and not evidence-based and may in fact be harmful” (p. 388).

### 3.13. The Updated NICE ME/CFS Guidelines

The British National Institute for Health and Care Excellence (NICE) published its updated ME/CFS guidelines in October 2021 [4]. It reviewed many CBT and GET trials and found that they were all of low or very low quality. Not one of them reached a higher level of quality. NICE also concluded that neither CBT nor GET lead to recovery nor were they effective treatments for ME/CFS. Additionally, GET is harmful and should not be used and CBT should only be used if patients have comorbid depression or anxiety or need help coping with this debilitating disease.

### 3.14. Statistical Issues

As noted by Kohl, a professor in medical statistics, in his response to the draft report [94], there are a number of statistical issues with the report. According to the report itself, there is a high risk of bias in all three included studies, but there are more methodological problems with these studies, so that the high risk of bias was a very high risk. (In German: “Es gibt sogar noch eine Vielzahl weiterer methodischer Einwände, die gegen diese Studien vorgebracht werden können und auch bereits vorgebracht wurden. Insgesamt kann man diesen Studien daher nur eine schlechte methodische Qualität zusprechen und muss nicht nur von einem hohen Risiko für eine Verzerrung an sich ausgehen, sondern, dass diese zudem recht groß sein könnte” p. 2). Professor Kohl also noted that it is difficult or almost impossible to do a meta-analysis based on just two studies (the analysis by the report was done using two studies for CBT and two for GET) especially because of the very low quality of the included studies. He also notes that “one must therefore ask oneself the much more fundamental question of whether these meta-analyses, which are based on only two studies of poor methodological quality, should have been carried out at all.” (In German, “Man muss sich daher im vorliegenden Fall die viel grundlegendere Frage stellen, ob man diese Metaanalysen, die nur auf zwei Studien von schlechter methodischer Qualität basieren, überhaupt hätte durchführen sollen” p. 2). In support of that, he noted that Egger et al. concluded that “if the ‘raw material’ is flawed, the findings of reviews of this material may also be compromised“ [94,95], because the results of a meta-analysis can only ever be as reliable as the studies on which they are based. (In German, “Die Ergebnisse einer Metaanalyse können immer nur so verlässlich sein, wie die Studien, auf denen diese basiert” p. 2). Moreover, if one does a meta-analysis, then there is the risk that statistical analyses give bad data an objective impression and thus lead to wrong conclusions. (In German, “Es besteht im Gegenteil sogar die Gefahr, dass statistische Analysen den schlechten Daten einen objektiven Eindruck verleihen und damit zu falschen Schlussfolgerungen verleiten” p. 2). The question also arises as to why, in the scenarios presented, that neither a highly probable overestimation of the effect nor a relevance threshold was taken into account in the calculations. This would change the results significantly and would mean that the necessary effects in the subpopulation without PEM would be far less extreme. (In German, “Außerdem stellt sich die Frage, warum bei den dort dargestellten Szenarien weder eine hoch wahrscheinliche Überschätzung des Effekts noch eine Relevanzschwelle bei den Berechnungen berücksichtigt wurden. Dies würde die Ergebnisse relevant verändern und würde dazu führen, dass die notwendigen Effekte in der Teilpopulation ohne PEM weit weniger extrem ausfallen müssten” p. 2, 3). The consequence of all these statistical problems would be to reject the results of these meta- and sensitivity analyses as unreliable. Under no circumstances should the IQWiG derive recommendations, neither in the current case nor in the future, from results that are highly likely to be distorted. (In German, “die Ergebnisse dieser Meta- und Sensitivitätsanalysen als unzuverlässig zu verwerfen. Keinesfalls sollte das IQWiG weder im aktuellen Fall noch in Zukunft aus mit hoher Wahrscheinlichkeit verzerrten Ergebnissen Empfehlungen ableiten” p. 3).

## 4. Discussion

The recently published draft report of the German IQWiG Institute recommends using CBT and GET for patients with mild and moderate ME/CFS. Their recommendation is based on two CBT (Janse et al. [24] and the PACE trial [20]) and two GET studies (the PACE trial [20] and the GETSET trial [30]). In this analysis we have highlighted several issues with those studies but also with the report itself. These issues include using a definition of CBT for ME/CFS by the report, which is not how CBT for ME/CFS is defined or was tested by the studies that were used. Consequently, those studies do not provide evidence for the efficacy of CBT because they did not test it in that form. 

The two studies that were used to test the efficacy of GET were both done by the same principal investigator (psychiatrist professor White). The fact that no GET studies were included from research teams from other institutes or from other parts of the world raises doubt about the reproducibility and accuracy of the findings of the two studies. Moreover, the GETSET trial [30] itself specifically states that it did not use GET. Janse et al. [24] tested the efficacy of two forms of Internet based CBT. It did not test the efficacy of face-to-face CBT which is the treatment recommended by IQWiG. Consequently, both studies should have been excluded from the analysis.

The report used the recently updated NICE ME/CFS guideline as its basis. That guideline excluded studies if less than 95% of patients suffered from the main characteristic of the disease which as concluded by NICE and the IQWiG report is PEM. The IQWiG report however changed less than 95% into less than 80% without explaining why. Consequently, up to 20% of participants could have a different disease. This is of particular concern because these patients did not suffer from PEM or exertion intolerance, in an analysis of exercise studies and CBT studies for ME/CFS that contain an element of exercise. The consequence of this is that that might lead to the erroneous interference of efficacy and safety for ME/CFS in its absence. The report also noted that 82% (GET) and 84% (CBT) of participants in the PACE trial suffered from PEM according to the study itself but that the study used an incorrect and too lax definition of PEM. Unfortunately, the report did not do anything with this conclusion even though the consequence of that conclusion is that the PACE trial overestimated the percentage of participants with PEM. Janse et al. used the Fukuda criteria as their only selection criteria. Most (97%) of the participants in Janse et al. fulfilled these criteria, and 92% of those suffered from PEM. The PACE trial and the GETSET trial used other criteria, but they also used the Fukuda ones. As we demonstrated in the article, if we apply the same ratio of 92% to the PACE trial and the GETSET trial then less than 64% of participants in these two studies suffered from PEM. Consequently, the report should have concluded that both studies did not fulfill their minimum requirement that at least 80% of participants in a study should suffer from PEM and both studies should have been excluded from the analysis. Why the report did not do this is unclear.

The report based its recommendation on a few subjective outcomes in non-blinded studies. For CBT it concluded there was a hint of a benefit at short and medium term for the outcomes fatigue, social participation and general symptoms and feeling sick after exertion (p. 149). Regarding GET it concluded that there was a hint of benefit for the outcomes “general symptoms” and “feeling sick after exertion”, derived from the results of the PEM survey, with medium-term indication of a benefit of GET. The report also concluded that CBT and GET had no relevant or statistically significant effect on the other subjective outcomes (p. 149, 150). Yet, basing a recommendation for both treatments on small improvements on a few subjective outcomes when they do not have any effect on the other subjective outcomes according to the report itself, is a form of reporting bias according to the IQWiG methods guidance handbook (p. 201). 

Additionally, according to the cognitive behavioral model, which forms the basis of using CBT and GET for ME/CFS, the reduction in physical functioning is fatigue related. This means that if the improvement in fatigue would be a real improvement, then physical functioning should improve accordingly. Yet according to the report, CBT led to a small improvement in fatigue but had no subjective or objective effect on physical functioning. This suggests that the improvement in fatigue is an artifact caused by all the biases of the studies. In the PACE trial, the fatigue scores at the end of treatment and after 52 weeks, were 21.5 and 20.3, respectively. Yet the fatigue entry score was 18 or more. This means that after CBT, most patients were still ill enough to enter the same trial and be treated with the same “effective” treatment again.

Moreover, it is unclear why the report based its recommendation on the improvement on a few subjective outcomes and ignored the null effect on the other subjective outcomes, such as it is unclear why it ignored the objective outcomes. This is especially puzzling since the IQWiG methods guidance handbook states that in non-blinded studies, an analysis should not rely on subjective outcomes but should use objective ones instead. This is also acknowledged by the authors of the GETSET trial [30] who stated that they do not actually know if their treatment led to real improvement because they did not use an objective outcome. The PACE trial, however, did use objective outcomes. CBT and GET did not lead to objective improvement according to the aforementioned six-minute walk test as noted by the report, and its step test scores and the occupational and benefit status did not improve either. Yet, even though the report acknowledged the absence of improvement on the six-minute walk test, this was ignored in the rest of the report. Why the report ignored this as well as the null effect on the other objective outcomes, is unclear.

Adherence to treatments, especially in the GETSET trial [30] and Janse et al. [24] was very low. According to the IQWiG methods guidance handbook, results should not be analyzed if more than 20 or 30% of participants dropped out. Up to 59% in the GETSET trial and 81% and 84% for the two treatment groups in Janse et al. [24], did not adhere to treatment. The percentage of participants who adhered to the treatment was therefore much lower than required according to the handbook because of that. Consequently, those two studies should have been excluded from the analysis. Why the report did not do this is unclear.

The report noted that the studies did not provide any evidence that the treatments are safe, yet it went on to conclude that CBT and GET are safe, because, according to the report, there was a tendency to a small subjective improvement in a few subjective outcomes. This not only ignores all the evidence that shows that these treatments are unsafe, but it also ignores the fact that 32% of treatments that are approved by regulating agencies, which did not report safety issues before they were approved, showed many issues with the safety of those treatments after approval, according to a review by Downing et al. [73]. One of the categories of treatments that was especially affected was psychiatric treatments. Moreover, one cannot assume a treatment to be safe based on the tendency of a small improvement on a few subjective outcomes in view of the (very) low adherence to the treatments in the three studies and the fact that the studies included up to almost 50% of patients who did not have PEM. Consequently, these patients did not have the disease in question, did not have any problems with incremental increases in exercise contrary to patients with ME/CFS. This led to an erroneous interference of safety by the report in its absence. On top of that, treatments that are harmful in up to 80% of patients and render up to 25% of patients bedridden, as found by a British University, contradict the *Do No Harm principle* of medicine, and should not be used, prescribed, or recommended. Medication with such a poor safety record would be withdrawn from the market with immediate effect.

Additionally, there were statistical issues with the studies because the risk of bias in the studies was very high and the methodological quality was very low. Moreover, the report based its CBT and GET recommendations on two studies each. Yet from a statistical point of view, one cannot produce a proper meta-analysis, let alone a high-quality one, based on just two studies because the results of such a meta-analysis and sensitivity analysis are likely to be too distorted to be reliable.

Finally, the British National Institute for Health and Care Excellence (NICE), stopped recommending these treatments in their updated guideline from October 2021. It also specifically noted that GET is harmful and should not be used and that CBT, in its original form as designed by Beck, should only be used if patients have a secondary depression or anxiety disorder or difficulties coping with this chronic debilitating multisystem disease. This conclusion has become even more relevant because millions of people have or are developing long COVID, which, in many cases, is the same or resembles ME/CFS, after an infection with SARS-CoV-2. Our analysis does not lend any support to assume that those treatments would be effective for those patients either.

## 5. Conclusions

There are many problems with the three studies that were used by the draft IQWiG report yet were ignored by the report. There are also several important issues with the report itself, as highlighted in our analysis. The IQWiG report used two studies each to investigate the efficacy of CBT and GET. These studies do not provide any evidence that CBT and GET are safe and effective. CBT and GET do not lead to an improvement in quality of life, reduction of the CFS symptom count or objective improvement, and they have a negative effect on the occupational and benefit status. On top of that, both CBT and GET are harmful and contradict the *Do No Harm principle* of medicine. Consequently, these treatments should not be used, prescribed or recommended for patients with ME/CFS. Our analysis does not lend any support to use those treatments for post-COVID syndrome, often referred to as long COVID, either, which, in many cases, is the same or resembles ME/CFS.

## Figures and Tables

**Table 1:** Percentage of patients with PEM, ME/CFS and percentage of misdiagnosed patients.

Studies	% with PEM According to the Studies	Fukuda Criteria	% with PEM as Defined by the IQWiG Report	% of Participants who Were Misdiagnosed
GETSET trial [30]	100%	68% (GES)	63% (GES)	37% (GES)
Janse et al. [24]	89% in both iCBT groups	97% (155/160 both iCBT groups) *	89% in both iCBT groups	11% in both iCBT groups
PACE trial [20]	84% CBT; 82% GET	62% CBT; 61% GET	57% CBT; 56% GET	43% CBT; 44% CBT

GES = guided graded exercise self-help. *: Five patients were included in the treatment groups with less than the four required additional symptoms.

## Data Availability

Not applicable.
